# Proteomics Standards Initiative at Twenty Years: Current Activities and Future Work

**DOI:** 10.1021/acs.jproteome.2c00637

**Published:** 2023-01-10

**Authors:** Eric W. Deutsch, Juan Antonio Vizcaíno, Andrew R. Jones, Pierre-Alain Binz, Henry Lam, Joshua Klein, Wout Bittremieux, Yasset Perez-Riverol, David L. Tabb, Mathias Walzer, Sylvie Ricard-Blum, Henning Hermjakob, Steffen Neumann, Tytus D. Mak, Shin Kawano, Luis Mendoza, Tim Van Den Bossche, Ralf Gabriels, Nuno Bandeira, Jeremy Carver, Benjamin Pullman, Zhi Sun, Nils Hoffmann, Jim Shofstahl, Yunping Zhu, Luana Licata, Federica Quaglia, Silvio C. E. Tosatto, Sandra E. Orchard

**Affiliations:** 1 Institute for Systems Biology, Seattle, Washington 98109, United States; 2 European Molecular Biology Laboratory, European Bioinformatics Institute (EMBL-EBI), Wellcome Trust Genome Campus, Hinxton, Cambridge CB10 1SD, United Kingdom; 3 Institute of Systems, Molecular and Integrative Biology, University of Liverpool, Liverpool L69 7ZB, United Kingdom; 4 Clinical Chemistry Service, Lausanne University Hospital, 1011 976 Lausanne, Switzerland; 6 Department of Chemical and Biological Engineering, The Hong Kong University of Science and Technology, Clear Water Bay, Hong Kong 999077, P. R. China.; 7 Program for Bioinformatics, Boston University, Boston, Massachusetts 02215, United States; 8 Skaggs School of Pharmacy and Pharmaceutical Sciences, University of California San Diego, La Jolla, California 92093, United States; 9 Department of Computer Science, University of Antwerp, 2020 Antwerpen, Belgium; 10 SA MRC Centre for TB Research, DST/NRF Centre of Excellence for Biomedical TB Research, Division of Molecular Biology and Human Genetics, Faculty of Medicine and Health Sciences, Stellenbosch University, Cape Town 7602, South Africa; 11 Univ. Lyon, Université Lyon 1, ICBMS, UMR 5246, 69622 Villeurbanne, France; 13 Bioinformatics and Scientific Data, Leibniz Institute of Plant Biochemistry, 06120 Halle, Germany; 14 German Centre for Integrative Biodiversity Research (iDiv), 04103 Halle-Jena-Leipzig, Germany; 15 Mass Spectrometry Data Center, National Institute of Standards and Technology, 100 Bureau Drive, Gaithersburg, Maryland 20899, United States; 16 Database Center for Life Science, Joint Support Center for Data Science Research, Research Organization of Information and Systems, Chiba 277-0871, Japan; 17 Faculty of Contemporary Society, Toyama University of International Studies, Toyama 930-1292, Japan; 18 School of Frontier Engineering, Kitasato University, Sagamihara 252-0373, Japan; 19 VIB-UGent Center for Medical Biotechnology, VIB, 9052 Ghent, Belgium; 20 Department of Biomolecular Medicine, Faculty of Medicine and Health Sciences, Ghent University, 9052 Ghent, Belgium; 21 Center for Computational Mass Spectrometry, Department of Computer Science and Engineering, Skaggs School of Pharmacy and Pharmaceutical Sciences, University of California San Diego, San Diego 92093-0404, United States; 22 Institute for Bio- and Geosciences (IBG-5), Forschungszentrum Jülich GmbH, 52428 Jülich, Germany; 23 Thermo Fisher Scientific, 355 River Oaks Parkway, San Jose, California 95134, United States; 24 National Center for Protein Sciences (Beijing), Beijing Institute of Lifeomics, #38, Life Science Park, Changping District, Beijing 102206, China; 25 Fondazione Human Technopole, 20157 Milan, Italy; 26 Department of Biology, University of Rome Tor Vergata, 00133 Rome, Italy; 27 Institute of Biomembranes, Bioenergetics and Molecular Biotechnologies, National Research Council (CNR-IBIOM), 70126 Bari, Italy; 28 Department of Biomedical Sciences, University of Padova, 35131 Padova, Italy

**Keywords:** Human Proteome Organization, mass spectrometry, proteomics, Proteomics Standards Initiative, molecular interactions, standards

## Abstract

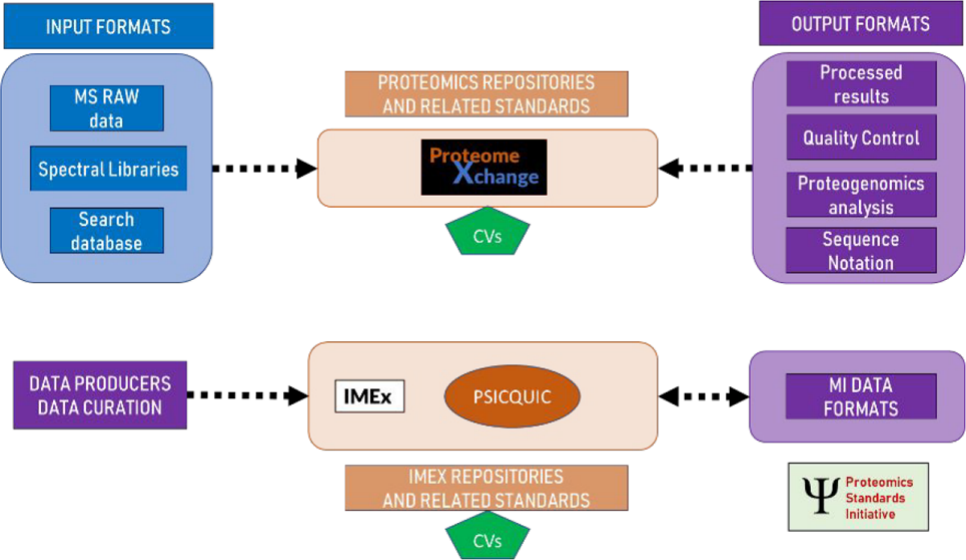

The Human Proteome Organization (HUPO) Proteomics Standards Initiative (PSI) has been successfully developing guidelines, data formats, and controlled vocabularies (CVs) for the proteomics community and other fields supported by mass spectrometry since its inception 20 years ago. Here we describe the general operation of the PSI, including its leadership, working groups, yearly workshops, and the document process by which proposals are thoroughly and publicly reviewed in order to be ratified as PSI standards. We briefly describe the current state of the many existing PSI standards, some of which remain the same as when originally developed, some of which have undergone subsequent revisions, and some of which have become obsolete. Then the set of proposals currently being developed are described, with an open call to the community for participation in the forging of the next generation of standards. Finally, we describe some synergies and collaborations with other organizations and look to the future in how the PSI will continue to promote the open sharing of data and thus accelerate the progress of the field of proteomics.

## Introduction

The field of proteomics has seen tremendous advances over the past 20 years, with the emergence of faster and more sensitive instruments, new acquisition workflows that collect more data on more ions per run, and more advanced software capable of better analysis on far greater volumes of data. The identification and quantification of a substantial fraction of the entire proteome of some species in a single mass spectrometry run is becoming feasible, facilitating the collection of information about protein abundances, molecular interactions, and protein functions at tremendous scale.^1,2^

Driving these advances is a diverse ecosystem of data analysis software packages, both from academic laboratories as well as from commercial companies. The benefit of such diversity is enhanced if there is also a robust set of standardized data formats that enables the interoperability of the software and also between software and bioinformatics data resources, such as the members of the ProteomeXchange^3−5^ and IMEx^6^ consortia, for MS proteomics and molecular interaction data, respectively. Although some data types are relatively simple such that *ad hoc* tab-delimited formats are sufficient, most proteomics data types are sufficiently complex that information-rich structured formats are necessary to avoid massive loss of metadata and provenance information. An important effort in the biomedical research field overall is to promote making all data findable, accessible, interoperable, and reusable (FAIR),^7^ and officially approved and recognized standards is a major component in the effort to make data FAIR.^8^

The Human Proteome Organization^9^ (HUPO) Proteomics Standards Initiative^10,11^ (PSI) was formed 20 years ago in 2002, under the leadership of Rolf Apweiler, Ruedi Aebersold, and others committed to the formation of an organization that would develop standards for the field of proteomics. At the time there were only vendor formats for raw mass spectrometry data and a few oversimplified plain text formats specific to the software tools of the time. Yet it was recognized that standardized formats would allow not just tool interoperability, but also promote the sharing and reuse of data between laboratories.

The mission of the PSI was, and still is, to bring together tool developers from academia, software vendors, and hardware vendors to create, maintain, and promote data standards that will be used throughout the proteomics and computational mass spectrometry community. These products include standardized data formats, minimum information guidelines, and controlled vocabularies (CVs) used to drive the formats. Standard formats are only effective if they are widely implemented in software tools, and thus the PSI also includes extensive outreach efforts and works with software developers to implement its standards. As a result, adoption and widespread implementation of PSI standards has enabled the development of software, such as Cytoscape, and APIs (Application Programming Interfaces), such as PSICQUIC^12^ and ProXI, which enable easy access to rich data streams by a broad spectrum of the research community.

In this article we first provide an overview of the operation of the PSI, highlighting its most recent workshops. We then describe the CVs, guidelines, and data formats that have been developed and ratified by the PSI over the years (summarized in Table 1). Next, we describe the set of standards that are currently in various phases of development, with an open call for participation by anyone willing to contribute to our efforts. Finally, we provide a brief discussion of synergies with other related organizations in the life sciences community and conclude with a vision of future contributions to the field.

**Table 1:** Summary of Standards, Reporting Requirements Documents and Controlled Vocabularies Released and under Development within PSI^a^

Working Groups	Guidelines	*v.*	Formats	*v.*	Controlled Vocabularies	*v.*
Molecular Interactions	MIMIx	1.1.2	PSI-MI XML	2.5.4	PSI-MI CV	2.5.0
	MIABE	1.0.0	PSI-MI XML	3.0.0		
	MIAPAR	1.0.0	MITAB	2.7, 2.8		
Mass Spectrometry	Mass spectrometry (MIAPE-MS)	2.98	mzML	1.1.0	PSI-MS	4.0.15
			TraML	1.0.0	XLMOD	1.1.0
Proteomics Informatics	Identification (MIAPE-MSI)	1.1	mzIdentML	1.2.0		
	Mass spectrometry Quantification (MIAPE-Quant)	1.0	mzQuantML	1.0.1		
			mzTab	1.0.0		
			mzTab-M	2.0.0		
			proBed	1.0.0		
			proBAM	1.0.0		
			PEFF	1.0.0		
			USI	1.0.0		
			ProXI (under development)			
			ProForma	2.0		
			mzSpecLib (under development)			
Quality Control			mzQC (PSI spec. under development)			
Protein Modifications					PSI-MOD	1.031.6
Intrinsically Disordered Proteins	MIADE (under development)					

a Links to documentation about each standard can be obtained from https://www.psidev.info/.

## Operation of the HUPO-PSI

The PSI is organized as a set of working groups (WGs) and an overall steering group. Each working group consists of a chair, one or two cochairs, and several named positions such as secretary, editor, guidelines coordinator, CV coordinator, and web content maintainer. In addition to this leadership group, each working group consists of other members contributing to the group, with substantial overlap in membership between the groups. The currently active working groups are the Mass Spectrometry, Molecular Interactions, Proteomics Informatics, Protein Modifications, Quality Control, Intrinsically Disordered Proteins, and Metabolomics Coordination Working Groups. The PSI Steering Group consists of an overall chair, two cochairs, a secretary, one or more editors, a guidelines coordinator, ontology coordinator, and Web site maintainer, plus all of the chairs and cochairs of the active working groups. The Steering Group generally meets monthly to coordinate working group activities, plan workshops, and develop outreach efforts. A summary of the roles within the organization and the current persons fulfilling those roles are documented at https://psidev.info/roles.

The general membership of the PSI is open to all who wish to participate. Everyone is encouraged to post to the corresponding mailing list or contact any one of the leadership members of a working group to indicate interest in a specific project, and be included in the ongoing activities. Organizations that have been in operation for so long can seem closed and cliquey, but the PSI actively seeks to dissuade this notion. Individual standards or projects are not necessarily led by working group chairs, but ideally led by those who are most interested in completing a project, regardless of their status. In general, the PSI only develops standards where at least one working group participant is an active champion of the standard and drives its forward progress. Proposed or desired standards without a champion usually are not developed. However, anyone in the community with the desire to see a new or updated PSI standard is very much encouraged to become a member of the PSI and champion that project within the umbrella of the PSI.

The PSI ratifies standards via a mechanism called the Document Process^13^ (DocProc). The DocProc is a formal process by which a proposed specification is thoroughly reviewed and refined before becoming an officially ratified standard of the PSI. Once a draft specification has been prepared by a working group, the process begins with the submission of a proposed specification to one of the PSI editors who has not been involved in the development of the specification. If the specification is deemed ready by the editor, it is sent to the Steering Group for a 14-day internal review period to assess initial suitability. Steering Group comments are then addressed by the proposers and the revision is resubmitted. Next, the editor selects at least two external reviewers familiar with the subject matter but not part of the development of the standard. The peer reviewers are generally anonymous, although there is precedent for reviewers requested to be listed in acknowledgments in recognition of their often-substantial time spent reviewing a specification. The specification is then revised based on the comments of the reviewers, after which the revision is subjected to a 4-week open community commenting period during which the proposal is widely advertised as a nearly complete standard and any additional comments from the community at large are sought. Ultimately when all comments received have been addressed to the satisfaction of the handling editor, the specification is declared ratified as an official PSI standard. The DocProc may be revised with the approval of the Steering Group, and has undergone several revisions in the past 20 years. The current version 1.1.2 is available at the PSI Web site at https://www.psidev.info/psi-doc-process. It is common that a journal article describing the standard in brief is prepared, submitted, and reviewed independently (ideally in parallel to the PSI review process) by a journal and generally, the standard is not declared ratified until both the DocProc review and journal review are complete.

Efforts to develop specifications continue all year at weekly calls and *ad hoc* meetings. Additionally, the PSI hosts a yearly workshop to bring everyone together to discuss the ongoing work in more depth. The workshops have traditionally been held during the March-May period each year in different locations throughout the world in an effort to attract new members in different regions. These workshops also have the effect of spurring extra progress in the weeks prior to and after the workshop.

## 2022 PSI Spring Workshop

The 2022 PSI Spring Workshop was held at the European Bioinformatics Institute (EBI) on the Wellcome Trust Genome Campus in Hinxton, United Kingdom, a fitting location for the 20th anniversary as the inaugural PSI workshop was also hosted at the EBI. This workshop was also the first in-person workshop in three years, since the SARS-CoV-2 pandemic caused the 2020 and 2021 workshops to be held fully online. Previous workshops were held in Cape Town, South Africa in 2019, at the European Molecular Biology Laboratory (EMBL) in Heidelberg, Germany in 2018, and at the National Center for Protein Sciences, Beijing (Phoenix Center) in Beijing, China in 2017. The 2020 workshop was originally organized for the University of California San Diego, California, USA, but was forced fully online due to the pandemic.

The 2022 workshop was a hybrid event with 31 in-person participants at the EBI, and 44 members participating via Zoom. The first day began with general overviews of progress and workshop plans by each working group plus an update by Juan Antonio Vizcaíno on the current state of the ProteomeXchange Consortium. After a short break, the Molecular Interactions Working Group split off into a separate track while the Mass Spectrometry, Proteome Informatics, and Quality Control Working Groups continued in a joint session to briefly discuss all of the topics relevant to all three working groups since there is substantial overlap in interests between the members of these groups. The second day was devoted to substantial progress on the formats mzSpecLib and mzQC (both discussed further below) in parallel tracks, while the MI group focused on format implementation and data curation. The third day began with more parallel track development and ended with a final plenary session with summaries of progress in each of the parallel tracks. The individual track sessions typically involve minimal presentation and mostly discussion of unresolved items that need group input, general planning, and development and assignment of action items.

## Controlled Vocabularies and Ontologies

An ontology is a collection of concepts with names and definitions and clear relationships between all the terms, typically with “is a” and “part of” relationships (e.g., a wheel is a part of a car, and a car is a vehicle, and a vehicle is a thing, although typically much more complex). A crucial component of the ontology is the relationships. A similar collection of concepts where the focus is on the concept names and definitions and not the relationships is typically thought of as CV. Thus far the PSI concept collections, widely used as proteomics-related ontologies,^14^ are part way between ontologies and CVs and are described further below.

The PSI-MI (Molecular Interactions) CV was first published in 2004^15^ and, since then, has been regularly extended to encompass new experimental methodologies and to serve extensions made to the formats to accommodate new data types. The CV is maintained on GitHub (https://github.com/HUPO-PSI/psi-mi-CV/blob/master/psi-mi.obo) and is readily available through the Ontology Lookup Service (www.ebi.ac.uk/ols/ontologies/mi) and BioPortal (https://bioportal.bioontology.org/ontologies/MI). An issue tracker on GitHub enables new term or update requests to be submitted by the user community. Use of the PSI-MI CV is an integral step in describing interaction data using the PSI-MI formats.

The PSI-MS (Mass Spectrometry) CV was initially developed to serve the needs of the PSI mass spectrometry-related data formats, most of which make extensive use of CV terms to precisely describe metadata and provide extensibility as technologies advance.^16^ The CV was first developed to support the now-deprecated mzData format and then for its replacement mzML,^17^ and has since been adapted for numerous additional formats that will be further described below. It contains over 3,300 terms for specific instrument models, software packages, and other metadata concepts and continues to grow as more such terms become available. This CV is readily accessible at https://github.com/HUPO-PSI/psi-ms-CV/ and is typically updated at least monthly. The contents of the CV is also browsable via the Ontologies Lookup Service^18,19^ (https://www.ebi.ac.uk/ols/ontologies/ms) and the NCBO BioPortal^20^ (https://bioportal.bioontology.org/ontologies/MS).

PSI-MOD is an ontology of modified amino acid residues.^21^ The 2,116 terms focus on the end products of modifications rather than modifications themselves. Thus, instead of terms for phosphorylation and oxidation, there are terms such as O-phospho-l-serine and l-methionine sulfoxide. The concepts are organized in several hierarchies by amino acid and by modification type. Although the ontology suffered from a period of inactivity several years ago, volunteers have now stepped forward to maintain it actively. The ontology is maintained on GitHub (https://github.com/HUPO-PSI/psi-mod-CV) or is readily available through the Ontology Lookup Service (https://www.ebi.ac.uk/ols/ontologies/mod). Complementary CVs, maintained by other organizations, which contain information on protein modifications include RESID,^22^ PTMList (https://uniprot.org/docs/ptmlist.txt), Unimod,^23^ and XLMOD.^24^ PSI-MOD was originally based on RESID, which is now mostly deprecated and used by very few tools. UniProtKB (UniProt KnowledgeBase) uses its own PTMList resource (https://uniprot.org/docs/ptmlist.txt), although PTMList is substantially synchronized with PSI-MOD. The popular Unimod community resource of mass modifications (http://unimod.org/) instead focuses on the modifications themselves (with terms like “Phospho” and “Oxidation”) and is a flat CV with no relationship structure.

The XLMOD CV (https://github.com/HUPO-PSI/xlmod-CV) is a simple collection of known chemical cross-linkers for use with formats that need to describe which cross-linker was used in the sample handling.

All these ontologies and CVs are maintained in the OBO format. However, each commit is versioned with a new minor release number and an OWL format file is autogenerated using the ROBOT conversion tool (http://robot.obolibrary.org/convert.html) via GitHub actions. Assistance with maintenance of the CVs and ontologies is a good entry point for those with proteomics domain knowledge who wish to begin contributing to the PSI.

## Guidelines

In addition to data formats and CVs, the PSI has also developed several sets of guidelines. These guidelines typically describe at minimum which pieces of information should be provided when releasing or sharing data, without specifying the encoding for that information. This minimum information may be provided in the text of an article, in an article’s Supporting Information, or in a PSI standard format. PSI standard formats are often designed with the aid of previously developed guidelines, ensuring that they are capable of capturing at least all of the minimum information and usually much more.

The first standard published was the Minimum Information about a Molecular Interaction eXperiment (MIMIx), which advises the user on fully describing a molecular interaction experiment, in either a publication or a database, and lists which information is important to capture.^25^ The document is designed as a compromise between the necessary depth of information to describe all relevant aspects of the interaction experiment, and the reporting burden placed on the scientist generating the data. The MIMIx standard has remained pertinent for over 15 years and the original publication remains the relevant documentation.

The Minimum Information About a Proteomics Experiment (MIAPE) guidelines^26^ were developed following the style of the Minimum Information About a Microarray Experiment (MIAME) guidelines^27^ for microarray experiments. The MIAPE guidelines were developed in a modular structure, such that each aspect of a proteomics experiment would correspond to a module, and the modules that pertain to a specific experiment could be used. Modules for the column chromatography^28^ (MIAPE-CC), the mass spectrometry^29^ (MIAPE-MS), the subsequent informatics analysis^30^ (MIAPE-MSI), and finally the quantitative components^31^ (MIAPE-Quant) of an experiment were developed. These MIAPE components were implemented in some systems such as the ProteoRed database^32^ and used as a guide in other software and during the development of formats. However, it seems that while most researchers would like others to provide rich metadata about their data sets, most researchers are reluctant when asked to provide all that information about their own data sets and avoid systems which require that they do so. In practice, the MIAPE guidelines have significant overlap with minimal reporting guidelines/checklists developed by individual journals (e.g., https://www.mcponline.org/mass-spec-guidelines for *Molecular and Cellular Proteomics* and http://pubsapp.acs.org/paragonplus/submission/jprobs/jprobs_proteomics_guidelines.pdf for the *Journal of Proteome Research*). These journals apply these guidelines to encourage or enforce that particular details are provided at least in Methods sections. Given the wide range of journals publishing proteomics data sets, it is likely an unsolvable problem to get all journals to sign up to a shared set of guidelines.

Although not formally ratified by the PSI, the PSI supported the development of the HUPO Human Proteome Project (HPP) Mass Spectrometry Data Interpretation Guidelines. Three such versions have been produced. The 1.0 version of these guidelines described the repository deposition requirement for data contributed to the HPP (https://hupo.org/HPP-Data-Interpretation-Guidelines). Version 2.1 of the guidelines added additional requirements for false-discovery rate (FDR) thresholding and for evidence supporting claims of detection of human proteins.^33^ Version 3.0 of the guidelines further refined these ideas to provide more stringent requirements for exclusion of false positives.^34^ Version 3.0 was published in 2019 and no further refinements seem necessary as of 2022.

## Existing Standard Data Formats

**Figure 1:**
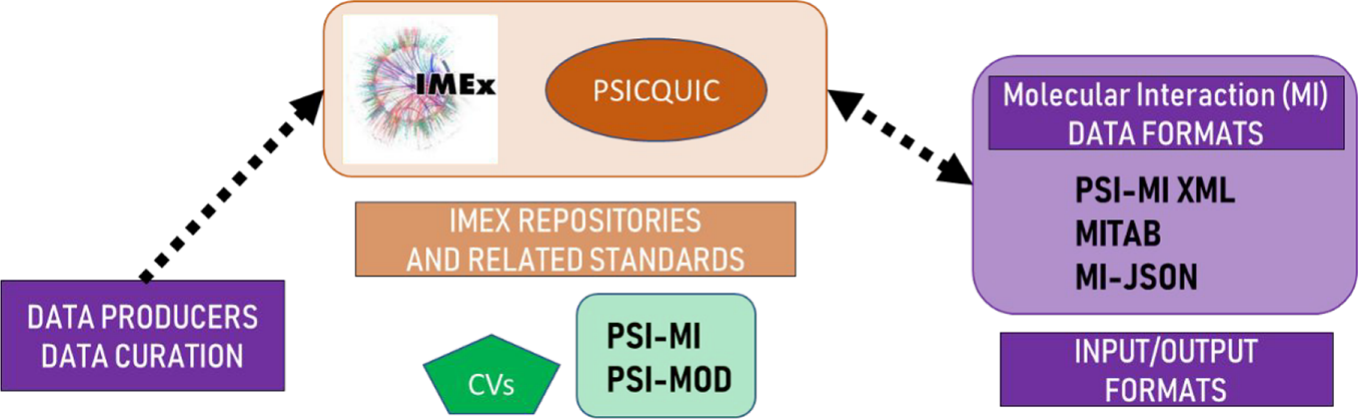
Overview of the formats developed by the Molecular Interactions Working Group and their relationships to other components in the community. Logo courtesy of IMEx.

**Figure 2:**
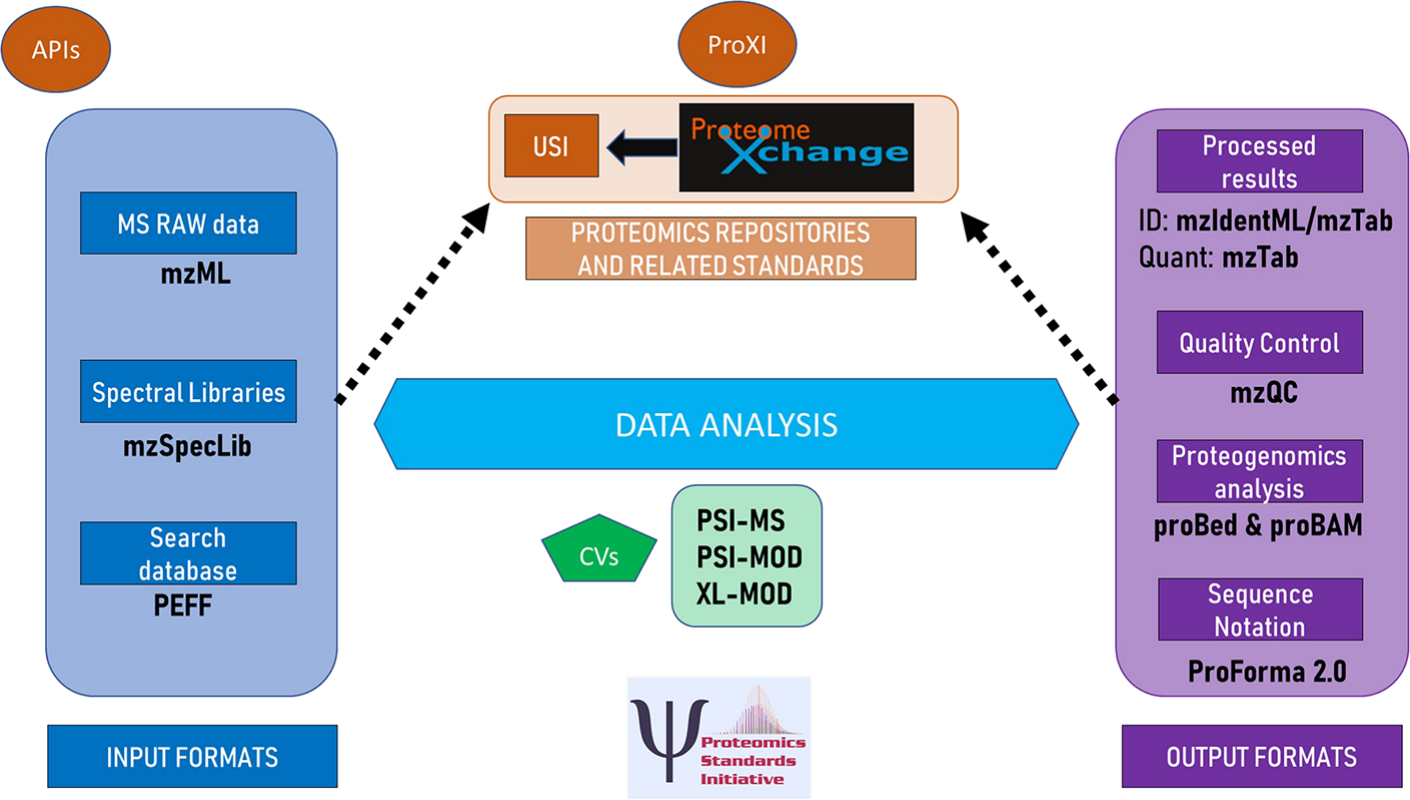
Overview of the formats of the Mass Spectrometry Working Group and the Proteome Informatics Working Group and their relationships to other components in the community. Logo courtesy of the Proteomics Standards Initiative and ProteomeXchange.

The PSI has developed numerous standardized data formats in the past 20 years and more are currently under development (Table 1). An overview of the formats developed by the Molecular Interactions Working Group and their relationships to other components is summarized in Figure 1. The formats of the Mass Spectrometry Working Group and the Proteome Informatics Working Group and their relationships to other components are summarized in Figure 2. In this section we briefly describe each format, assess its current status, and provide links and citations for obtaining more information. Active formats are presented by working group, followed by non-PSI related formats and deprecated formats.

### Molecular Interactions Working Group

#### PSI-MI XML

The MI group released an XML standard (PSI-MI XML) in 2004,^15^ which allowed the basic information about a protein–protein interaction experiment to be captured and transferred between data resources or visualization/analysis tools. This initial, very simplistic representation was upgraded in 2007 to enable a full capture of the details of any experiment, including *in silico* and predictive data, which describes an interaction between any number of biomolecules, including experimental details, molecule details (affinity tags, binding sites, amino acid mutations, etc.), and the host organism in which the experiment is undertaken. Additional information, such as kinetic parameters or the method by which a molecule is delivered or engineered into a cell can also be added, if required.^35^ The PSI-MI XML 2.5 version of the format remains the main workhorse by which the majority of interaction data is exchanged between resources, and enables all of the above use cases. As more specialist use cases have arisen which could not be fully captured in this format, in 2018 a new, backward compatible version, PSI-MI XML 3.0, was developed.^36^ This version can be used to describe, for example, the details of a fully curated multiprotein complex with subunit topology, including binding regions, and stoichiometry or to link kinetic parameters to amino acid mutations, sequence deletions or insertions.

#### MITAB

Following requests from bench scientists for a simpler version of the PSI-MI XML format for use by researchers not experienced in working with XML files, the MITAB format was developed to enable most aspects of a molecular interaction experiment to be described. The format shares usage of the PSI-MI CVs and many fields are identical to those in the XML formats, but the data is described in tab-delimited format with increasing number of columns in the different versions (MITAB 2.5, 2.6, 2.7, 2.8) allowing increased data capture. The most recent version, MITAB 2.8 also referred to as CausalTAB, enables the representation and dissemination of signaling information through the description of the causality of an interaction.

#### MI-JSON

MI-JSON is the recommended protocol for serving interaction data to web pages and visualization tools. The format is described at https://github.com/MICommunity/psi-jami/blob/master/jami-interactionviewer-json/schema/mi-json-schema.json.

#### Java Software Library JAMI

JAMI^37^ is a single Java library and framework which unifies the standard formats such as PSI-MI XML, PSI-MITAB, and MI-JSON as well as formats not created by the PSI. Adopting JAMI avoids conversions between different formats and avoids code/unit test duplication as the code becomes more modular. The JAMI model interfaces are abstracted from each format to hide the complexity/requirements of each and enables the development of software and tools on top of this framework (https://github.com/MICommunity/psi-jami).

### Mass Spectrometry and Proteomics Informatics Working Groups

#### mzML

The primary standardized format for encoding the output of mass spectrometer instruments is mzML.^17^ All instruments write out their primary data in a binary vendor format. Most vendors provide software libraries to read their data files, but these are often only available on the Microsoft Windows platform. In order to facilitate data analysis on any platform, and ensure that data files could always be read, the PSI developed the open XML-based format mzML. Version 1.0 was released in 2008 and a minor fix version 1.1 was released in 2009; version 1.1 has been stable and widely used ever since.

The focus of mzML has always been universal readability, and thus small file size and fast access speed have not been the primary design drivers. As a result, numerous alternatives to mzML that improve on file size and access speed have been proposed,^38−41^ although none seem to have gained substantial usage, perhaps in part because of their dependencies or complexity. A variant of mzML called imzML^42^ has become quite common in the imaging MS community, and it is nearly identical to mzML except that the spectra are stored in a more efficient sidecar file instead of the main file. In order to try to consolidate on one improvement over mzML, the PSI is considering formally approving the HDF5-based mzMLb format^43^ as a PSI standard in addition to mzML. The mzMLb format has exactly the same schema and encodes metadata in the same way, and thus interconversion is easy. Via its use of HDF5, which automatically incorporates compression, file sizes are much smaller than mzML and random access to spectrum within the file is also much faster. Adding mzMLb to existing tools is relatively straightforward if the HDF5 dependency can be included.

#### Ongoing Work: mzML Extension for DIA and IMS Data

As described above, the mzML format has been stable and widely used since 2009. At the time, data independent acquisition (DIA) and ion mobility spectrometry (IMS) were not widely used and not explicit factors in the design. Now that they have become widely used, there is great interest in extended mzML for use with these technologies. Fortunately, the schema definition does not need to be changed, and good support for these technologies can be achieved with some additional CV terms and an explicit best practices document that describes how these types of data should be encoded in mzML. This best-practices document has been drafted and is nearly ready for submission to the DocProc. The current draft is available at the mzML web page (https://psidev.info/mzml/).

#### mzIdentML

The primary PSI standard format for encoding the peptide/protein identifications that are derived from an MS proteomics experiment is mzIdentML. The version 1.1, considered as the first stable version, was released in 2011^44^ and version 1.2^45^ in 2017, adding features for workflows such as *de novo* sequencing, cross-linking, proteogenomics approaches and improved encoding of protein inference results. The mzIdentML format is widely implemented in over 35 software tools (https://www.psidev.info/tools-implementing-mzidentml) and an encouraged (but not required) format at the ProteomeXchange data repositories, since it is one of the standard formats that can be used for performing “Complete” submissions (those where the identification data can be parsed and linked to the mass spectra by the receiving repository).

#### Ongoing Work: mzIdentML Extension for Glycopeptide and Cross-Linked Peptide Data

The mzIdentML format still does not fully meet the needs for some special workflows, and efforts are underway to define best practices for improving the encoding of more complex arrangements of cross-linked peptide identifications and to improve the representation of glycopeptide identifications. Although not supported in the first stable 1.1 version of mzIdentML, initial support for cross-linked peptide identification data was added in mzIdentML version 1.2. However, it has been determined that some more complex features of cross-linking data, e.g., working with cleavable reagents, are not satisfactorily encoded. In addition, there is an effort underway to develop more extensive documentation and best-practice guidelines for encoding glycopeptides as well.

In both cases, this will not require a schema change, but rather, the development of best practice documents. CV terms will be used to define how these data types should be encoded in a consistent manner. Participation is actively sought.These enhancements are expected to be completed in 2023.

#### mzTab

Although mzIdentML has been widely adopted, it was felt that for some applications, a simpler tab-delimited format capable of encoding the most important information would be beneficial. The mzTab format^46^ was conceived as such a relatively simple format that could encode both identification and quantification, applicable to both bottom-up MS proteomics as well as small-molecule MS metabolomics. It has been implemented in several software packages (mainly for identification data, not for quantification) such as Mascot,^47^ OpenMS,^48^ MaxQuant,^49^ and ProteomeXchange repositories.

After some years of use, it was concluded by PSI working groups that while the attempt to support both proteomics and metabolomics in the same format was a noble idea, the result was a format that did not serve either data type as well as desired. As a result, mzTab-M^50^ was redesigned as a format that would fix some perceived design issues and support only metabolomics. Its data model is backed by a JSON-based schema, supporting serialization into a tab-separated main storage format or JSON as a transfer format. Since then, it has been adopted by a number of metabolomics and lipidomics software packages and repositories (https://github.com/HUPO-PSI/mzTab#current-activities-and-software-support). Additionally, similarly to mzIdentML, mzTab can also be used for performing “Complete” submissions to ProteomeXchange data repositories.

Preliminary discussions have started to produce a similarly redesigned mzTab-P 2.0 for proteomics only, based on the experience of the design of mzTab-M, although to date the updated format has not progressed rapidly and hence participation in this design process is actively sought.

#### proBAM and proBed

The proBAM and proBed formats^51^ are relatively straightforward adaptations for proteomics of the tab-delimited BAM/SAM and BED formats widely used in genomics. Existing columns were defined to be applicable to peptide-based MS proteomics, and a few additional columns were added for peptide-specific contexts. Peptide data thus written in proBAM and proBed are compatible with transcript alignment viewers and other similar software already widely used in transcriptomics. No further changes are planned. Unfortunately, the formats have not been widely adopted so far. Most people use their own variation of the original BED format for representing peptide coordinates in a genome context, by using a small number of columns. Additionally, there is not a perceived need yet to use the more verbose proBAM format. In our view, by using these formats, proteogenomics studies would benefit from a greater standardization in data representation. The added advantage is that most genomics viewers developed would be able to visualize these files.

#### PEFF

The PSI Extended FASTA Format^52^ (PEFF) was developed as a format that is broadly compatible with the ubiquitous FASTA format, but defines mechanisms for file-level metadata, multiple sequence collections within a file, collection-level metadata, and how a wide variety of additional information can be encoded on a per-sequence basis. Whereas the description lines in FASTA files are free form and vary widely based on the data provider, the format of description lines for PEFF files are defined in the specification and can be extended with controlled vocabulary terms. This allows PEFF files to encode proteins and their modifications, such as post-translational modifications (PTMs), protein processing such as signal peptides, and sequence variants. Implementations of PEFF support in Comet^53^ enables transparent searching for known PTMs and sequence variants.^54^ Although it was not originally designed with this use case in mind, PEFF can also be used to represent proteoforms, as detailed in section 3.4 of the PEFF specification.

#### ProForma 2.0

The Consortium for Top-Down Proteomics (CTDP) defined the initial 1.0 ProForma notation^55^ to encode exact proteoforms with all applicable PTMs on a specific sequence. When the PSI needed a mechanism to encode in a compact way exact peptidoforms (peptide sequences with a specific set of mass modifications), ProForma provided a useful piece of prior work on which to build. In collaboration with the CTDP, the PSI has recently developed the ProForma 2.0 standard,^56^ which provides a substantially expanded set of mechanisms to encode a wide variety of modified proteins and peptides (proteoforms and peptidoforms) in a manner that meets the needs of both the CTDP as well as the bottom-up proteomics workflows. The ProForma 2.0 formatting is both easily human readable as well as software parsable. The ProForma 2.0 standard is used in conjunction with other completed and in-development PSI standards as described below.

#### Universal Spectrum Identifier (USI)

There are numerous cases when one or more mass spectra should be carefully examined since they provide crucial evidence for a scientific conclusion. Often such spectra are published as figures in journal articles or may appear in Supporting Information as static figures that prevent close scrutiny. Additionally, in the context of FAIR data, it is recommended to have unique identifiers for different types of entities (in this case mass spectra) in public data repositories. While not a file format in itself, the PSI has defined the USI^57^ as a multipart key that can be copy-pasted into manuscripts or other conversations about important spectra in order to facilitate the identification and retrieval of specific spectra and PSMs. USIs currently identify spectra that have been deposited into one of the ProteomeXchange public data repositories, although extensions are underway to support USIs for spectra in spectral libraries, both for proteomics as well as metabolomics. If the USI includes an optional proposed interpretation, it is encoded using ProForma 2.0 notation. See http://proteomecentral.proteomexchange.org/usi for proteomics examples, and https://metabolomics-usi.ucsd.edu/ for metabolomics examples.^58^

### Related Formats

Although not officially ratified formats of the PSI, there are a few formats that are highly related to the efforts of the PSI and ProteomeXchange, and will be briefly described here.

#### ProteomeXchange XML (PX XML)

The ProteomeXchange (PX) Consortium^3−5^ has been receiving and making public proteomics data sets from the community since 2012, and PX members are highly active in the PSI. The data sets themselves always remain at the receiving repository for a submission, but the most important metadata about each data set is transmitted from the receiving repository to ProteomeCentral (http://proteomecentral.proteomexchange.org/) whenever a data set is made public. Searches involving public data sets in any ProteomeXchange resource can then be enabled. The common format for this is PX XML (current version is 1.4, http://proteomecentral.proteomexchange.org/schemas/proteomeXchange-1.4.0.html), which encodes study metadata such as title, description, submitter, publication, location of availability, submitted files, etc. The format is similar to mzML and mzIdentML in basic design and since its development in 2011 has evolved to include an increasing amount of metadata as ProteomeXchange requirements increase.

#### MAGE-TAB for Proteomics (SDRF-Proteomics and IDF)

Although the PX XML format provides substantial study-level metadata and a list of files submitted with the study, it does not provide for specific sample metadata and a mechanism to link individual files to specific samples of the study. The MAGE-TAB for Proteomics format,^59^ which is an extension of the original MAGE-TAB format used in transcriptomics,^60^ has been recently adapted by ProteomeXchange resources to capture the sample metadata and the experimental design for proteomics experiments. MAGE-TAB for proteomics has two main components: the Investigation Description Format (IDF) and the Sample and Data Relationship Format (SDRF-Proteomics). The MAGE-TAB-Proteomics files are compatible with the original transcriptomics versions, but several adaptations/extensions are included for the proteomics use case.^55^ IDF is quite analogous to the PX XML format, although it is less structured and is not as rich in information as PX XML. PX XML files can be easily converted by the ProteomeXchange resources into IDF with some loss of information.

SDRF-Proteomics files provide the previously missing—and much needed—mechanism for sample-specific annotations with CV terms, and encode the links from those samples to the submitted data files. Sample attributes are encoded in the columns of the files, where the column definitions are required to be CV terms and the data values are either CV terms or plain scalar values when CV terms are not appropriate. Several ProteomeXchange repositories now accept and process SDRF-Proteomics files upon submission of data sets, and the GitHub repository at https://github.com/bigbio/proteomics-metadata-standard provides a mechanism for anyone in the community to manually curate and generate SDRF-Proteomics files for ProteomeXchange data sets that were previously released. The MAGE-TAB for Proteomics effort was primarily driven by the EuBIC-MS group, in collaboration with the PSI. As mentioned above, submission of IDF files is not required since all the information required to create them is made available at submission time.

### Disused Formats

Not all formats developed by the PSI have been successful and widely used. The mzData format released in 2005 was deprecated in 2008 with the release of mzML, which incorporated all of mzData’s functionality. The sepML and gelML formats^61^ were well designed and still potentially usable, but there was no interest in the community to encode the many details of gel-based workflows and for other separations used in proteomics experiments and have been deprecated due to little interest. Should interest revive in capturing such data, they remain good foundational work. The TraML format^62^ for encoding selected reaction monitoring (SRM; also called MRM, multiple reaction monitoring) transition lists and DDA inclusion lists was used by a few software packages^63^ after its release, but the extreme popularity in the SRM field of the Skyline software,^64^ which used its own .sky format and never supported TraML, rapidly led to the TraML format becoming largely unused.

mzQuantML was developed as an XML-based format to capture a detailed output of proteomics quantitative workflows.^65^ However, its adoption was limited due to a preference from most software developers to export quantitative output data in flat text files. When mzTab was released later, there was the hope that this simpler tab-delimited format could become popular to represent the final results coming from quantitative experiments. As mentioned above, although mzTab has been implemented, it has been mainly used for capturing identification results only so far. The field has been unable to agree so far on a format for capturing quantitative information. This situation hinders further progress and the reuse and integration of proteomics data.

A clear lesson is that PSI formats are most successful when there are a variety of tools with developers who want the standardized format and participate in its development, ideally along the entire data life-cycle from data acquisition, preprocessing, identification, quantification to statistical analyses, visualization, and eventually data deposition and publication.

### New Standards in Development

Most of the formats mentioned thus far have completed the standardization process, are complete, and in use. However, the PSI is actively working on several new formats that are in various stages of the standardization process. In all cases, community involvement is always being sought, either for the design phase, early adoption in software or for the review phase. Readers with interest in any of the subsequent formats are encouraged to contribute to their development and ratification.

#### mzQC

The qcML format^66^ was published in 2014 as an XML PSI-like format for encoding mass spectrometry quality control (QC) metrics, but without the PSI ratification process. However, several shortcomings were soon identified and general interest in QC waned, both of which hindered adoption. Renewed interest for a QC format within the PSI has led to a simplified, yet more versatile JSON-based new format, called mzQC, that fixed these shortcomings and includes the participation of members of the community most interested in supporting mzQC in their QC tools.^67^ As a result, many QC-relevant concept and metric terms have been proposed for integration into the PSI-MS CV. This JSON-based format is currently under review in the PSI DocProc.

#### mzSpecLib

There have been several spectral library formats in wide use in the community for some time, including the NIST (National Institute of Standards and Technology) MSP format, the SpectraST^68,69^ speclib format, several versions of the blib format,^70^ the hlf format, ELIB and DLIB (https://bitbucket.org/searleb/encyclopedia/wiki/EncyclopeDIA%20File%20Formats), etc. While any one of these formats does a good job in encoding the spectra in the library, there is general agreement that none of the formats do a good job in encoding the metadata associated with the library in a consistent manner.^71^ An example is MSP, wherein nearly all metadata is recorded within the COMMENT field in a highly variable manner across software packages. Various producers and consumers of spectral libraries have come together to create a new standard called mzSpecLib (https://psidev.info/mzSpecLib). It is quite similar to the MSP and speclib formats, with an emphasis on heavy use of CV terms and a standardized data model that can be encoded in several different serialization mechanisms, including an MSP-like text format, a JSON format, and potentially a more performant binary format based on HDF5 or SQLite. Spectrum interpretations are encoded using ProForma 2.0 and references to spectra external to the library (e.g., the contributors to a consensus spectrum) are encoded with USIs. The mzSpecLib specification is expected to enter the PSI DocProc in 2023.

#### mzPAF

Although originally conceived and developed as part of mzSpecLib, a standardized format for encoding fragment peak annotations has been split into a separately proposed standard (see details at https://psidev.info/mzSpecLib). This was done in order to keep the mzSpecLib specification smaller, because the annotations for other types of molecules, such a lipids and glycans, would be quite different but envisioned as an add-on to mzSpecLib, and finally because a standardized annotation format may be useful in other contexts, such as in spectrum viewers and figures containing annotated spectra. The formatting is based substantially on the NIST MSP peak annotation formatting, which has evolved over the years but was never documented. Several components differ from NIST MSP conventions by general consensus, with participation by NIST as well. This mzPAF peak annotation format is about to enter the DocProc.

#### ProXI

The partners of the ProteomeXchange consortium have been developing an API for the communication of proteomics data called ProXI, formally the ProteomeXchange eXpression Interface. The API enables the programmatic access to information about data sets, proteins, peptides, peptidoforms, peptide-spectrum matches (PSMs), and spectra via a consistent interface for all ProteomeXchange partners as well as an aggregator at ProteomeCentral. ProXI remains a work in progress, with several of the end points designed and at least partially implemented at several partners. Due to the extensive software development that must still take place, dedicated funding will likely be required to complete the design and achieve implementations at all ProteomeXchange resources. ProXI is the mechanism that drives the multirepository USI lookup and display mechanism at ProteomeCentral described above. ProXI uses the OpenAPI 3.0 platform for designing end points with a JSON communication format. The data set end point is modeled after the PX XML schema, and the spectrum end point is modeled using the mzSpecLib schema described above. The current state of the ProXI schema definition is found at https://github.com/HUPO-PSI/proxi-schemas.

#### PTM Site Formats

As part of “PTMeXchange”, a project funded by the United Kingdom Biotechnology and Biological Sciences Research Council (BBSRC) and the United States National Science Foundation (NSF) to improve sharing and deposition of high-quality sets of PTMs, a set of formats to encode PTM results are being developed. These are intended to be as simple as possible while still encoding sufficient information to properly evaluate FDR values and provide transparent validation information such that the results can be filtered to have low false positives and be transferred to knowledge bases such as UniProtKB. The set of formats is envisioned to include a PSM-level format, a peptidoform-level format, and a site-level format. The design is in progress with initial drafts already available, and further participation from the community is welcome.

#### MIADE

The Minimum Information About Disorder Experiments (MIADE) guidelines aim to provide a standard to improve the reproducibility, interpretation, and dissemination of data generated by the Intrinsically Disordered Proteins (IDP) field. The guidelines provide recommendations for data producers on how to describe the results of their IDP-related experiments, for biocurators on how to annotate experimental data in manually curated community resources, and for database developers on how to disseminate the data. Information about the protein region, the structural state, and the experimental and computational approaches is required to create a MIADE-compliant description of an IDP experiment.

The PSI-IDP working group drafted and submitted for publication an article^72^ describing the MIADE guidelines and will submit the full specification to the PSI document process as well. The PSI-IDP working group also implemented these guidelines in DisProt, a manually curated resource of intrinsically disordered proteins and regions from the literature.^73^ With the adoption of the MIADE guidelines, DisProt biocurators can now provide more detailed and comprehensive annotation by including information about the sequence construct, the experimental conditions, and experimental components.

## Future Work and Synergies with Other Organizations

The PSI develops and ratifies standards as part of the community and as such attempts to foster synergistic activities with other organizations within the community in order to maximize its impact. As one of the long-standing HUPO initiatives, the PSI undertakes development efforts that further the mission of HUPO, its other initiatives, and major projects such as the HPP. An important example is the participation in the development of the HPP MS Data Interpretation Guidelines (versions 1, 2.1, and 3.0) as described above. These guidelines are not formally a PSI product, but were developed with the extensive experience of PSI members. As also highlighted above, the PSI cooperates extensively with the ProteomeXchange and the IMEx Consortia, which both actively use and promote the PSI standards.

Although primarily focused on proteomics, the PSI does reach out to, and has members from the metabolomics and lipidomics communities to extend the use of PSI standards for metabolomics and lipidomics applications, with substantial success for mzML and mzTab-M, and good future perspectives for USI and mzSpecLib. In this context, the PSI also fosters collaboration with computational-focused groups, such as the CompMS group (https://compms.org), which promotes the development of computational mass spectrometry algorithms and training in their use. The CompMS group includes participation from both proteomics and metabolomics researchers who use MS.

The PSI also collaborates with the European Bioinformatics Community for Mass Spectrometry^74^ (EuBIC-MS) (https://eubic-ms.org/), which promotes development of MS bioinformatics tools and provides training on how to apply them. As a concrete collaboration, the EuBIC-MS group was leading the development of the MAGE-TAB for Proteomics format.

The Global Alliance for Genomics and Health^75^ (GA4GH) is a policy-framing and technical standards-setting organization, enabling the responsible sharing of clinical and sensitive genomic data through both harmonized data aggregation and federated approaches. The discussion about whether human sensitive proteomics data should be subjected to the same access restrictions as sensitive DNA/RNA sequencing data has just started.^76,77^ The PSI will then follow closely the developments of the GA4GH since clearly, some existing standards could be adapted or extended to support proteomics data, if needed. In our view, definitely, it would not make sense to “reinvent the wheel”.

## Conclusion

We have presented here an overview of the most important aspects of the PSI after 20 years of efforts, including its current operation, the current status of existing standards, ongoing work for standards in progress, and synergies between the PSI and community groups. These activities demonstrate the commitment of the PSI in accelerating the pace of biomedical research by facilitating the dissemination and reuse of data and interoperability of software. There are many ongoing standards in development, and it is perhaps worth reiterating that greater community participation is always welcome, since such greater participation leads to better standards. As the PSI completes these standards, new needs will emerge as the field advances. Already there is growing interest in standardization around the rapidly emerging high-throughput affinity-based protein quantification platforms, and some initial discussions have taken place at PSI workshops, although a clear plan has not yet emerged.

The adoption of PSI standards has clear benefits in making proteomics data more FAIR (findability, accessibility, interoperability, and reusability), but since the PSI formats are comprehensive and complex, there is a substantial development cost over using *ad hoc* simple tabular formats. One clear direct benefit of the use of PSI standards is that “Complete” submissions to ProteomeXchange repositories using PSI standards is more FAIR. This means in practice that the files can be automatically parsed and the corresponding information can be linked to the mass spectra and as a key point, potentially to other bioinformatics resources (interoperable), and then traced back to the original experimental source (making it findable). Without standard formats, this becomes completely infeasible unless data reanalysis is performed. But this is far from ideal, because data reanalysis is a very resource-intensive activity, and also, because at least a subset of the original results is “lost” when using other tools, making the data as originally analyzed untraceable.

However, proteomics data are complex, and therefore comprehensive PSI formats are also complex. This means that the implementation of a PSI format such as mzIdentML is not an easy or short task, even for expert developers. Additionally, the existence of many different workflows (e.g., cross-linking and glycoproteomics) makes it very challenging in practice for software developers to support the formats. And finally, the dynamic nature of proteomics experimental approaches and the long time it takes for data standards to support novel approaches can sometimes make the standards seem constraining. Nonetheless, the PSI promotes the use of its standards by the progressive advancement of requirements in ProteomeXchange repositories and by working with journal editors to promote repository deposition requirements.

The PSI has developed a relatively large number of different standards, supporting different use cases, and indeed there are many other formats in use in proteomics that have not been developed by PSI, but are still useful in some contexts. There have been some sentiments expressed in the community that the PSI has developed too many different formats. This is usually countered by observing that most PSI formats cover different aspects of a very complex process, and trying to develop a single format that covers all aspects of proteomics would lead to an overly complex standard that would not be widely implemented on account of extreme complexity. While there are some examples highlighted above where PSI standards have not been widely used and hence deprecated, the ethos of the PSI is to make life easier, not more complex, for proteome scientists and bioinformaticians, and we continue to review our processes to ensure that new standards are only developed where there is a strong use case.

The PSI will thus continue its efforts to maintain and enhance existing standards to meet emerging needs. While many of the current PSI standards focus primarily on data-dependent acquisition workflows, more efforts are needed to apply standardization to DIA workflows.^78^ As software development leads to ever greater automation and artificial intelligence, the PSI should continue to foster the development and implementation of APIs and interchange systems that allow intelligent agents and software to interoperate in ways so that end-users need not concern themselves with which standards are being used, but rather rely on an ecosystem of formats, APIs, and software that allows them to focus only on their science.
